# Dietary Advanced Glycation End Products Consumption as a Direct Modulator of Insulin Sensitivity in Overweight Humans: A Study Protocol for a Double-Blind, Randomized, Two Period Cross-Over Trial

**DOI:** 10.2196/resprot.4552

**Published:** 2015-07-29

**Authors:** Barbora de Courten, Maximilian PJ de Courten, Casper G Schalkwijk, Karen Z Walker, Josephine Forbes

**Affiliations:** ^1^ Monash Centre for Health Research & Implementation School of Public Health and Preventive Medicine Monash University Melbourne Australia; ^2^ Centre for Chronic Disease College of Health and Biomedicine Victoria University Melbourne Australia; ^3^ Laboratory for Metabolism and Vascular Medicine Experimental Internal Medicine Maastricht University, Maastricht 6211 LK Netherlands; ^4^ Department of Nutrition and Dietetics Monash University Melbourne Australia; ^5^ Mater Research Institute- The University of Queensland Glycation and Diabetes TRI South Brisbane Australia; ^6^ Mater Clinical School School of Medicine University of Queensland St Lucia Australia

**Keywords:** advanced glycation, diet, type 2 diabetes, insulin sensitivity, insulin secretion, inflammation, carboxymethyllysine

## Abstract

**Background:**

Advanced glycation end products (AGEs) are formed during the processing, storage, and cooking of foods. As part of a western diet, AGEs are consumed in excess and impair glucose metabolism in patients with type 2 diabetes. In the absence of diabetes, AGE-mediated decreases in insulin sensitivity and signaling have been postulated. However, randomized studies to test this relationship in humans are limited.

**Objective:**

The primary aim of this trial is to determine whether dietary consumption of AGEs will decrease insulin sensitivity in healthy overweight adults. A secondary aim is to determine the effects of dietary AGEs on insulin secretion, circulating soluble receptor for AGEs (sRAGE), and inflammation markers.

**Methods:**

Overweight, but otherwise healthy, non-diabetic adults (N=20) aged 18-50 years old will complete a randomized cross-over design intervention study alternating low and high (4-fold increase) AGE diets (2-week duration). At baseline, participants will undergo a medical review including an intravenous glucose tolerance test (IVGTT), a hyperinsulinemic-euglycemic clamp, and anthropometric measures and questionnaires assessing diet, physical activity, and general wellness. Each test diet will be followed for 14 days, followed by a 4-week washout period before commencement of the second alternate dietary period. Energy, macronutrient, and AGE intake will be calculated for each dietary period. Additionally, the AGE content of foods used in the study will be measured by ultra performance liquid chromatography mass spectrometry. All measurements will be repeated at the beginning and end of each dietary period. Primary and secondary outcomes will be expressed as a change over the dietary period for insulin sensitivity, secretion, anthropometric parameters, sRAGE, and inflammation markers and compared by paired *t* test and analysis of variance (ANOVA).

**Results:**

The study will be completed in early 2016.

**Conclusion:**

The proposed trial will provide much needed clinical evidence on the impact of excess dietary AGE consumption on insulin sensitivity and will indicate whether lowering dietary AGE intake can improve insulin sensitivity and/or secretion, thereby decreasing risk for type 2 diabetes.

**Trial Registration:**

Clinicaltrials.gov NCT00422253; https://clinicaltrials.gov/ct2/show/NCT00422253 (Archived by Webcite at http://www.webcitation.org/6ZXLhT89c)

## Introduction

In both developed and developing countries, the consumption of highly processed foods has increased dramatically over the past 30 years [1]. This change in diet has been associated with increased exposure to advanced glycation end products (AGEs), which are formed in foods by processes such as non-enzymatic browning (Maillard reaction). AGEs and Maillard reaction products are important for flavor and color and increase the shelf-life of treated foods [2]. While foods high in sugar and protein are most susceptible to AGE formation, long-term storage, heating, and physical or chemical processes may also produce AGEs even in foods regarded as healthy such as fruit juice, milk, and cereals. In addition, foods high in fat and sugar can also facilitate in vivo formation and deposition of AGEs within tissues [3,4]. Cooking temperature with promotion of surface browning is also a critical factor: baking, roasting, frying, and grilling are potent promoters of advanced glycation [5]. High levels of AGEs are thus found in many common foods such as heated milk and other dairy foods, baked breads, biscuits and cookies, toasted breakfast cereals, grilled steak, brewed beer, and roasted coffee beans.

Type 2 diabetes is a global health problem and in many developed countries it has already reached epidemic proportions over the past few decades [6]. There is accumulating evidence from animal studies indicating that high dietary AGE consumption contributes to increased insulin levels, insulin resistance, defects in first phase insulin secretion, and type 2 diabetes [7-9]. Interestingly, low AGE diets can protect against declining insulin sensitivity and the onset of type 2 diabetes in animal models, even in the context of high fat intake and marked weight gain [7,8]. This suggests that it is the high AGE content in high fat diets and not the fat content per se, as was previously thought, that impairs glucose metabolism. In addition, successive generational feeding of a high AGE diet to rodents results in descendants with increased adiposity, insulin resistance, impaired insulin signaling, and a proinflammatory phenotype [10], which further implicates dietary AGEs in the etiology of type 2 diabetes.

In humans, one clinical trial demonstrated an association between the low dietary consumption of AGEs and improvement in insulin sensitivity in patients with type 2 diabetes [11]. Similar results were seen in another trial in people without diabetes [12], although macronutrients were not matched in these studies. Both trials used a homeostasis model assessment (HOMA-IR), which is an indirect measure of insulin sensitivity. HOMA-IR is unable to reliably differentiate between the insulin sensitivity and insulin secretion (HOMA-β) because both are calculated from fasting glucose and insulin concentrations. No studies have used gold standard measurements of insulin sensitivity and secretion to investigate the metabolic effects of high and low AGE diets. In particular, there is a paucity of data testing the effects of dietary AGEs on insulin secretion in both animals and humans. We have recently shown in rodents that changes in insulin secretion following long-term exposure to AGEs [13,14] can be prevented with AGE-lowering therapy [13]. In addition, there is a suggestion that an AGE-RAGE interaction could mediate β-cell failure from cell [13,15] and animal studies [16].

To date, there have been no human trials investigating the impact of a dietary AGE intervention on direct measures of insulin sensitivity and insulin secretion or on the development of type 2 diabetes. Therefore, our aim is to compare the effects of high and low AGE diets, followed for 2 weeks, on direct measures of insulin sensitivity and insulin secretion in healthy yet overweight or obese individuals without diabetes. We also plan to assess the contribution of AGE receptors and chronic low-grade inflammation to the changes in insulin sensitivity and secretion observed during the trial.

## Methods

### Study Design and Setting

#### Overview

**Figure 1:**
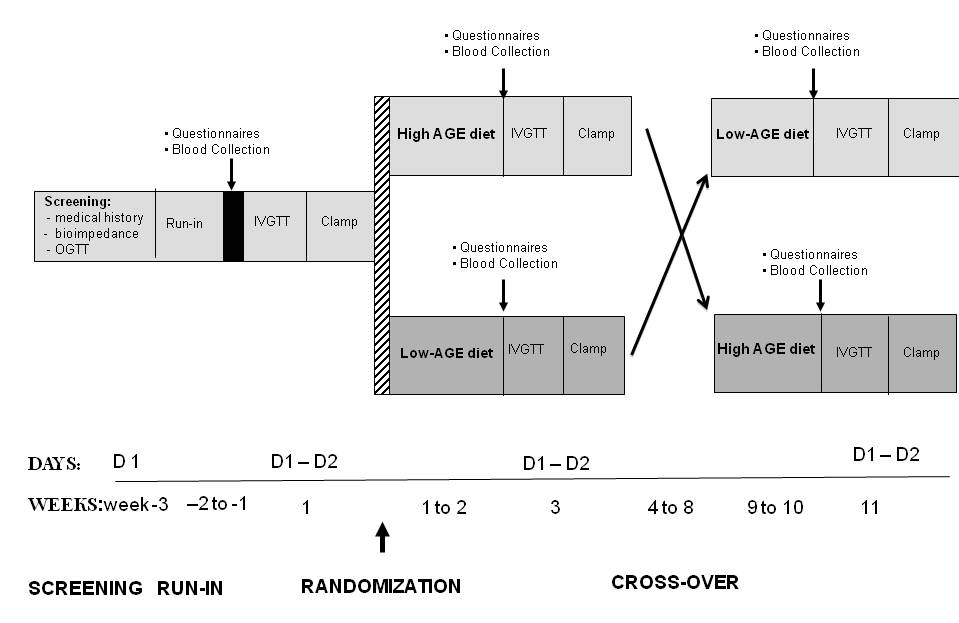
Trial protocol.

This study has a randomized, two period double-blind cross-over design (Figure 1). We aim for 20 overweight but otherwise healthy normoglycemic adults, aged 18-50 years to complete the trial. Participants will commence the study after a 2-week run-in on their habitual diet, but with restricted intake of alcohol, fast food, and coffee. Test diets will then each be followed for 2 weeks, separated by a 4-week washout period (return to habitual diet). Usual levels of physical activity will be continued throughout the study. Overweight participants will be selected for enrolment in this study as they represent the average Australian population, are generally more insulin resistant, more sedentary, and have higher levels of inflammatory markers, all factors that increase their risk for type 2 diabetes [17,18]. Participants will be sought using a number of advertising strategies including posters, flyers, newspaper, and online advertising and email newsletters from the Alfred Hospital in Melbourne, Australia.

#### Inclusion Criteria

Inclusion criteria for the study will include the following (1) aged 18-50 years, (2) free of diabetes (previously diagnosed or on the basis of the screening OGTT), (3) generally healthy upon medical screening, (4) overweight or obese but not morbidly obese (body mass index (BMI)>25 kg/m^2^ but <40 kg/m^2^), and (5) stable body weight, having exhibited a weight change <5 kg in the preceding year with no intention to lose weight or change physical activity during the trial.

#### Exclusion Criteria

Exclusion criteria will include (1) substance abuse including smoking and high alcohol use (>4 and >2 standard drinks per week for males and females, respectively), (2) known allergies, (3) use of medications including vitamin supplements and hormonal contraceptives, (4) any renal, cardiovascular, hematological, respiratory, gastrointestinal, endocrine or central nervous system diseases, psychiatric disorders, active cancer within the preceding 5 years, or the presence of acute inflammation or infection based on the medical history and the physical and laboratory examinations obtained at recruitment, and (5) women in menopause, or pregnant and/or lactating.

### Statistical Considerations

#### Sample Size Calculation

The sample size is estimated on the basis of the primary hypothesis including being able to detect a 20% difference in insulin sensitivity following dietary AGE modification using G*Power, an online tool to compute statistical power analyses. This effect size is clinically significant and similar to that seen after a 6-month intervention period using troglitazone, an insulin sensitizing therapy. Troglitazone improved insulin sensitivity as measured by glucose clamp in obese, non-diabetic individuals [19] whose initial glucose disposal rate was 8.1 (+2.0) mg/kg/min. To detect a 20% difference in insulin sensitivity in a cross-over design by paired *t* test, we will require complete data from 20 individuals (power 80%, alpha 5%, SD 1.0).

#### Data Analysis

Descriptive analysis will be performed on baseline characteristics and covariates. We will use paired *t* test to determine the significance of the change in measured parameters during test diets. Multiple regression will be used to assess the determinants of insulin secretion after adjusting for covariates. Dietary order will be included in multiple regression models as a between participant variable. Carryover effects will be tested in an expanded model including diet and period interaction when evaluating the effects of diet over the 2 test periods. Period effects refer to a change between intervention and measurement periods and within subjects, which would have occurred independently from any diet given. Plausible period effects in diet-based trials might arise from behavioral changes by participants as a result of enrolment in a dietary-based trial

Adjustment to *P* values for multiple comparisons will be performed using the Holm method, and statistical significance will be assumed when *P*<.05.

### Screening

The study timelines are presented in Figure 1. Female participants will be required to commence metabolic testing while they are in the follicular phase of their menstrual cycle and a urine pregnancy test will be performed to exclude pregnancy. At visit 1, screening will be undertaken by a registered medical practitioner who will collect the medical history and perform a physical examination including measurement of blood pressure, height, body weight, and waist and hip circumference using the methods outlined in the following sections.

At the second visit, participants will undergo an OGTT with measurement of fasting and 2-hour glucose levels to exclude diabetes according to the World Health Organization (WHO) 1999 criteria [20]. Blood samples will be analyzed by a single accredited laboratory for full blood count, kidney and liver function, lipid profile, and C-reactive protein (CRP) as a marker of inflammation. Participants will also be given a 3-day food diary to record their habitual dietary intake and this will be returned on their next visit where it will be used to aid the dietitian in the development of individualized test diets (as following).

### Baseline Assessment

Baseline assessments will commence at the third visit which will involve measurement of blood pressure and body composition by bioimpedance. At the fourth and fifth baseline visits, an intravenous glucose tolerance test (IVGTT) and a hyperinsulinemic euglycemic clamp will be performed (detailed below in Data Collection and Analyses). Participants will complete the International Physical Activity Questionnaire (IPAQ) questionnaire to assess habitual physical activity [21].

### Randomization and Blinding

Following successful screening and baseline assessment, subjects will be randomized by a computer program to commence the study with either the high or low AGE diet. Randomization will be done in blocks of four by gender using a relevant computer program to ensure balance in each test group and will be conducted by a researcher from within our department who is not involved in the trial data collection, analysis, or reporting. Diet type will be coded as (red or blue) by the dietitian, so that participants and blinded investigators remain unaware of which dietary period they are undertaking or how diet might affect glucose metabolism. Clinical and laboratory investigators will also be masked to diet allocation.

### Intervention

Using Australian food composition data in addition to data on the AGE, N-carboxymethyllysine (CML), N-carboxyethyllysine (CEL), and methylglyoxal-derived hydroimidazolone (MGH1) content of common foods [3], a set of carefully matched alternative food choices has been designed with each alternate food choice having similar macronutrient and total energy content but differing greatly in AGE (referred to as the "Food List") [9].

To ascertain dietary AGE content, food items used in the study were obtained from local supermarkets and prepared according to the instructions of the study. To prevent neo-formation of CML during acid hydrolysis, a reduction step with sodium borohydride was used. Food items containing <20% fat were mixed and subsequently deproteinized with 1000 µl cold (4°C) TFA. Food items >20% fat were mixed and subsequently deproteinized with a mixture of chloroform:methanol (2:1, v/v) [22]. After centrifugation (4300 *g*, 4°C, 20 min), the supernatant (TFA) or lower phase (chloroform) was carefully removed. For validation experiments, 25 µL of a standard solution (six point calibration curve; 5250-0 nmol/L CML, 6250-0 nmol/L CEL and 14749-0 nmol/L MGH1) was added. Samples were then hydrolyzed by adding 500 µl 6N HCl and incubated for 16 hours at 100°C. After hydrolysis, 40 µL hydrolysate and 20 µL internal standard (containing 1432 nmol/L ^2^H2-CML, 1378 nmol/L ^2^H4-CEL, and 1322 nmol/L ^2^H3-MGH1) were mixed in a reaction vial. The mixture was evaporated to dryness under a stream of nitrogen gas at 70°C. To increase sensitivity during electrospray ionization and increase retention time using reversed phase chromatography, samples were subsequently derivatized with 100 µL 1-butanol:HCl (3:1, v/v) for 90 minutes at 70°C. After derivatisation, the samples were evaporated to dryness under nitrogen, redissolved in 300 µL water, mixed, and subsequently centrifuged at 20,000 *g* for 20 minutes. AGE-free adducts in foods were measured.

Using the 3-day diet diary recorded by each participant during the "run-in" period, the dietitian will help participants select foods for their first test diet from the "Food List", guided by their individual preferences and energy requirements. The content of their second test diet will comprise the alternate paired foods. Food choices on the high-AGE diet thus will approximate a typical Australian diet, while the isoenergetic low AGE diet, matched for macronutrient content, will have reduced AGE content through altered cooking techniques [23] and use of differently processed foods. All food required for both test diets will be provided weekly to the participants as ready-to-eat items or as packed food portions together with detailed instructions for storage and re-heating or cooking (method, temperature, duration). Participants will be asked to eat to appetite throughout both dietary periods to maintain constant body weight and to complete a daily record indicating the number of portions eaten for each food item supplied, any foods not consumed, or any additional foods that were eaten.

Participants will be contacted twice a week by the dietitian who designed the dietary protocol to resolve any problems and to ensure dietary compliance.

### Follow-Up Visits

Participants will be scheduled for their follow-up visits after completing each test diet for 2 weeks. All the procedures performed during baseline assessment including blood pressure, anthropometry, IVGTT, physical activity questionnaire, and glucose clamp will be repeated with the exception of the OGTT, as this will only be performed once at screening to identify undiagnosed diabetes.

### Safety Considerations

During the screening, baseline, and follow-up procedures, any medical conditions or abnormalities detected will be promptly discussed with the participant by a qualified medical practitioner involved in the study. Where applicable, participants will be treated, referred, and/or advised to visit their general practitioner for follow-up. All participants will be advised of the results of their medical review and blood tests after completion of the study, and a medical practitioner from the study team will provide them with strategies to improve their diabetes and cardiovascular risk profile.

### Ethics

This trial has received ethical approval from the Alfred Hospital Ethics Committee in Melbourne, Australia (Protocol ID: 36/06).

### Outcome Measurements

The primary outcome in this trial is the difference (change) in insulin sensitivity between the 2 diets. Secondary outcomes include changes in insulin secretion, body weight, body mass index (BMI), waist, waist-to-hip ratio (WHR), resting systolic and diastolic blood pressure, lipid profile and markers of inflammation (ie, interleukin (IL)-1ß, IL-6, IL-8, and IL-10), tumour necrosis factor-α (TNFα), macrophage migration inhibitory factor (MIF), monocyte chemotactic protein-1 (MCP-1), C-reactive protein (CRP), nuclear factor-κB (NF-κB) activity, and circulating sRAGEs.

### Data Collection and Analyses

#### Anthropometry

##### Body Mass Index (BMI) and Percent Body Fat

Body weight (kg) and height (cm) will be measured using a digital scale (BC-418MA, Tanita UK Ltd) and stable stadiometer (Seca 206), respectively, at baseline and following the intervention period during which participants will be lightly clothed and without shoes. BMI will be calculated as weight (kg)/height (m)^2^. Body composition will be determined by bioelectrical impedance analysis (BC-418MA, Tanita UK Ltd).

##### Waist-To-Hip Ratio (WHR)

Central adiposity will be assessed using waist and hip circumferences, taken in duplicate by an experienced researcher using a constant-tension tape. Waist circumference will be measured at the midpoint between the upper iliac crest and the lowermost rib at the end of a normal expiration, while hip circumference will be taken around the widest part of the buttocks. The WHR will be determined as waist (cm)/hip (cm).

#### Metabolic Measures

All metabolic testing will be performed after a 12-hour overnight fast. Prior to metabolic testing, participants will be asked to abstain from strenuous exercise and caffeine for 3 days. The first metabolic testing day in females will be scheduled in the follicular phase of their menstrual cycle.

##### Oral Glucose Tolerance Test (OGTT)

Participants will ingest 75 g of glucose over 2 minutes. Blood samples will be drawn at 0 and 120 minutes to analyze plasma glucose levels and to determine diabetes status (WHO 1999 criteria).

##### Intravenous Glucose Tolerance Test (IVGTT)

Using IVGTT, acute insulin secretory response will be measured. First, baseline blood will be collected at -10 and 0 minutes, after which 50 ml of 50% glucose will be delivered intravenously over a 3-minute period. Blood will then be collected for measurement of insulin at the 3, 4, 5, 6, 8, 10, 15, 20, 25, and 30 minute time points. The early insulin secretory response will be calculated as the mean incremental plasma insulin level from the 3rd to the 5th minute after the glucose bolus.

##### Hyperinsulinemic Euglycemic Clamp

A euglycemic glucose clamp will be used to measure insulin sensitivity. After collecting baseline blood and plasma glucose levels at 0 minutes, the clamp will be initiated by an intravenous bolus injection of insulin (9 mU/kg). Insulin will then be constantly infused at a rate of 40 mU/m2/min for approximately 120 minute into an arm vein, whilst glucose is variably infused to maintain euglycemia. Plasma glucose values will be monitored every 5 minutes during the clamp while the variable infusion rate of glucose is adjusted to maintain blood glucose at a constant concentration of 5 mmol/L for the last 40 minutes of the clamp.

Plasma glucose concentrations will be measured by the glucose oxidase method (ELM 105 Radiometer). Plasma insulin levels will be measured by chemiluminescent microparticle immunoassay and plasma high sensitivity CRP will be determined by immunotubimetric assay (Abbott Archicentre ci162000).

#### Measurement of Advanced Glycation End Products (AGEs) and Their Receptors

The concentrations of AGE- (CML, CEL, and MG-H1) modified proteins in serum and free AGEs in urine will be quantified using UPLC MSMS as previously described [24]. AGE-modified proteins will also assayed [25] in urine (neat) using an indirect ELISA as previously described [26].

The pool of sRAGE (circulating RAGE from proteolytic cleavage and AGER gene transcription; RnD Systems) and endogenous secretory RAGE (esRAGE) (circulating RAGE from AGER1 gene transcription only) will be analyzed in plasma by ELISA according to the manufacturer’s instructions.

#### Cardiovascular Measures

##### Blood Pressure

Resting systolic and diastolic blood pressure and pulse rate will be measured using an automated oscillometric measurement system (Omron) after a 30 minute rest. The mean blood pressure derived from 3 measurements will be recorded.

##### Lipid Profile

Lipid profile-related parameters to be measured include plasma total cholesterol, triglycerides, and low density lipoprotein (LDL) and high density lipoprotein (HDL) cholesterol using a standard commercial enzymatic assay (Beckman Coulter LX20PRO Analyzer and Synchron) and Systems Lipid and Multi Calibrators (Beckman Coulter Diagnostics).

#### Inflammatory Markers

##### Cytokines and Chemokines

Plasma inflammatory markers (IL-1ß, -6, -8 and -10, TNFα, MIF, and MCP-1) will be measured using a commercial automated chemiluminescent enzyme immuno assay (EIA) and immulite analyzer (Diagnostic Products Corporation), while plasma CRP will be analyzed using highly sensitive near infrared particle immunoassay rate methodology and a Beckman Coulter Synchron LX system Chemistry Analyzer (Beckman Coulter Inc).

##### Nuclear Factor-κB (NF-κB) Activity

Nuclear extracts of white blood cells will be obtained and analyzed for the binding capacity of the p50/p65 subunit of NF-κB to an NF-κB oligonucleotide consensus sequence as per the manufacturer’s instructions (Active Motif, CA, USA).

#### Self-Reported Measures

##### Nutrient Analyses

During the test diets, the daily records of all foods eaten will be collected weekly from study participants. Food intake will then be analyzed for nutrient content by a dietitian using an Australian Food Composition program. Based on these records, the dietary intake of CML, CEL, and MGH1 will be determined for each participant over each dietary period. The mean daily energy, macronutrient, and AGE intake of participants during the 2 test diets will then be compared.

##### International Physical Activity Questionnaire (IPAQ)

The validated IPAQ determines the type of everyday physical activity that people engage in [21]. We will use the short version of IPAQ as a timely, convenient method to determine whether study participants have made any change in their physical activity which could influence our study outcomes. The short IPAQ asks participants to reflect on the past 7 days and report time spent on vigorous activity (eg, aerobics), moderate activity (eg, carrying light loads), walking, and sitting [21].

## Discussion

### Principal Findings

To the best of our knowledge, there is no human clinical trial published investigating the effect of high and low AGE diets on insulin sensitivity and secretion in individuals without diabetes, employing gold standard measures of insulin sensitivity and secretion, and comprehensively investigating mechanisms including chronic low-grade inflammation.

Insulin resistance increases with obesity and is a key pathogenic process underpinning type 2 diabetes. Interventions that reduce insulin resistance such as lifestyle measures including diet and exercise resulting in weight loss, as well as pharmacological therapies are used to prevent and treat type 2 diabetes [27]. However, to date, these agents have failed to decrease the burden of this disease. Diabetes is a major cause of morbidity and mortality, primarily due to chronic complications including an increased risk of cardiovascular disease. It is vital that additional effective primary prevention strategies are established to reduce insulin resistance and prevent and manage type 2 diabetes [28]. The current trial should thus inform and advance this important field of research.

By demonstrating that intake of a low AGE diet improves insulin sensitivity and/or secretion, large scale interventions using low AGE diets could potentially become a mainstream strategy for diabetes prevention in overweight and obese individuals. This strategy would offer a cost-effective and easily administered intervention that could have a considerable impact on health outcomes in Australia and worldwide since it can be achieved by simple changes in cooking methods by individuals and/or processing by the food industry. Such improvements will not only lower diabetes risk, but since AGEs are also known to play an essential role in the development of micro-vascular complications, a low AGE dietary intervention could reduce cardiovascular risk factors as well as chronic low grade inflammation [11]. Therefore, lowering AGEs could have beneficial effects on decreasing risk factors associated with a wide range of metabolic conditions. High-quality clinical trials such as this are, therefore, an important first step towards elucidating the effects of a low AGE diet in promoting health and well-being by improving insulin sensitivity, decreasing cardiovascular risk factors, and potentially decreasing the risk of type 2 diabetes and its associated micro and macro vascular co-morbidities.

There is a paucity of human data investigating the effect of AGEs on insulin resistance in humans. One previous study by Uribarri et al [11] examined the effect of dietary AGE restriction on glucose homeostasis in 18 diabetic and 18 healthy individuals. They showed that in patients with type 2 diabetes, but not in healthy individuals, insulin resistance (estimated by HOMA-IR) improved after 4 months of dietary AGE restriction. We have recently published another trial in overweight and obese people without diabetes that demonstrated an improvement of HOMA-IR with a low AGE dietary intervention when compared to a high AGE diet [12]. In both of these studies, the intake during the 2 diets was isocaloric but not macronutrient matched. Therefore, some of the changes observed could have been due to differences in macronutrient intake. In addition, these studies depended on HOMA-IR, which is calculated from fasting glucose and insulin levels and hence cannot differentiate between insulin sensitivity and secretion. No human trials with AGE dietary manipulation have been carried out to date measuring insulin secretion, but we and others have previously shown that a greater exposure to AGEs either at very high concentrations or for an extended duration may ultimately impair pancreatic beta cell function resulting in reduced insulin secretion, particularly of the first phase in cells and in animal models [13,15]. Such effects appear to be mediated by changes in the AGE receptor RAGE [13,14,16]. A few previous studies have suggested that cooking methods, which result in lower AGEs in the food, can alter glucose homeostasis, but these studies used either single meal challenges [29-31] or did not account for changes in body weight [32].

With regard to putative mechanisms involved, AGE modulation could impact on insulin sensitivity via effects on insulin signaling pathways [33,34], likely mediated by a chronic low-grade inflammation [35]. Consistent with this, in the study by Uribarri [11], a diet low in AGE content was associated with a decrease in circulating concentrations of inflammatory cytokines in patients with type 2 diabetes but not healthy individuals.

Recent reports have also demonstrated a possible role of another AGE receptor AGE-R1 in the development of insulin resistance in the type 2 diabetic population [11]. In the study by Uribarri, a diet low in AGE content was associated with an elevation in AGE-R1 expression on peripheral blood mononuclear cells (PBMC)s in patients with type 2 diabetes but not healthy individuals [11].

### Methodological Considerations

The strengths of the proposed study protocol include the gold standard study design, randomization, and double-blinding of both participants and investigators to limit bias. Other major strengths include the cross-over design which controls for individual variation between participants, the assessment of confounders such as dietary macronutrient and energy content and physical activity, and the use of direct rather than indirect measures of insulin sensitivity and secretion such as the OGTT, IVGTT, and the gold standard euglycemic clamp. Determination of many blood components will also allow for the comprehensive exploration of potential mechanisms involved. We will also have a comprehensive analysis of the dietary AGEs which are consumed during the study period which will be matched with AGE levels in both the blood and urine.

Despite these strengths, there are also potential limitations. First, self-selection bias may be present given that recruitment is based on voluntary participation by interested subjects. These subjects may not represent the entire target population because they may be characteristically different to other volunteers (ie, potentially more health conscious). Secondly, we are only recruiting overweight or obese adults (BMI>25), who are otherwise healthy (not medicated and without diabetes etc), and therefore the results of the study will not be generalizable to other populations such as those within a healthy weight range or those with diagnosed diabetes or other medical conditions and co-morbidities. Third, our study will only measure risk factors such as blood pressure, fasting glucose, and insulin sensitivity and insulin secretion. Longitudinal follow-up studies would be necessary to ascertain if change in these risk factors translates into decreased incidence of type 2 diabetes.

### Conclusions

Type 2 diabetes and its associated complications are associated with significant morbidity and mortality as well as healthcare costs. Insulin resistance, the key risk factor for type 2 diabetes, increases with increasing body weight. While existing research suggests that consumption of a low AGE diet may prevent insulin resistance and type 2 diabetes, well-designed randomized trials are lacking. If our clinical trial shows that low AGE diet can improve risk for diabetes, our study may have important public health implications and could lead to feasible and cost-effective strategies to prevent type 2 diabetes and its complications.

## Abbreviations

AGE: advanced glycation end product
BMI: body mass index
BP: blood pressure
CEL: carboxyethyllysine
CML: carboxymethyllysine
CRP: C-reactive protein
CVD: cardiovascular disease
DXA: dual-energy x-ray absorptiometry scan
ELISA: enzyme-linked immunosorbent assay
HbA1c: hemoglobin A1C
HDL/ LDL: high-/low-density lipoprotein cholesterol
HOMA-IR: homeostasis model assessment for insulin resistance
IL: interleukin
INFγ: interferon-gamma
IPAQ: International Physical Activity Questionnaire
IU: international units
IVGTT: intravenous glucose tolerance test
MCP-1: monocyte chemotactic protein 1
MGH1: methylglyoxal-derived hydroimidizolone
MIF: macrophage migration inhibitory factor
NF-κB: nuclear factor-κB
NHMRC: National Health and Medical Research Council
OGTT: oral glucose tolerance test
sRAGE: soluble receptor for advanced glycation end products
TG: triglycerides
TNFα: tumour necrose factor-α
WHR: waist-to-hip ratio

## Appendix

Multimedia Appendix 1 NHMRC assessment.
