# Supplementary material for: Dietary Advanced Glycation End Products Consumption as a Direct Modulator of Insulin Sensitivity in Overweight Humans: A Study Protocol for a Double-Blind, Randomized, Two Period Cross-Over Trial
Source: JMIR Res Protoc. 2015 Jul 29;4(3):e93. doi: 10.2196/resprot.4552 (PMC4750465; doi:10.2196/resprot.4552)
Supplement: Multimedia Appendix 1 [file resprot_v4i3e93_app1.pdf]

ONHMRC - GrantNet

ASSESSMENT REPORT FOR APPLICANT

Application Id: 586655 **Chief Investigator A:** Dr Barbora de Courten **Application Year :** 2009

Title: Will a reduction in dietary AGEs improve insulin sensitivity and secretion in overweight individuals?

Assessor 1

Scientific Quality

The proposed studies are of a high scientific quality and follow on from the studies in rats performed by CIB. The study is relatively well controlled and geared towards the primary endpoint of insulin sensitivity. The main concern with this project is how the investigators chose the list of low grade inflammatory markers and whether these are just observations. Considering the biological variability of such markers or surrogate endpoints, how will the investigators achieve anything scientific from these secondary endpoints?

Significance and Innovation

Contemporary Australian diets have a high content of advanced glycation end products (AGEs) due to industrialized methods of food processing with excess fat and high sugar content. The formation of AGEs via the Maillard reaction is thought to decrease insulin sensitivity. This study aims to investigate, in a cross over design whether a low AGE diet can increase insulin sensitivity in obese individuals. This project is clinically very important considering the massive burden of obesity on the economies of Developed Nations, and the associated morbidity and mortality. Albeit a small proof on concept study; a positive outcome may lend itself to larger efficacy trials.

Track Record in Relation to Opportunity

This is an excellent team of investigators with solid track records in the proposed study area. CIA has a very good track record with approximately half of her publications in leading journals. CIB has an excellent track record in the field of diabetes complications, is a JDRF CDA fellow, and leads a very successful laboratory. CIC and CID both have good track records

Budget

The budget as detailed is appropriate.

Questions for the Applicant

How have the investigators chosen the specific inflammatory markers?
The study is underpowered to analyse inflammatory markers. How will these data add scientific merit to the current proposal?

Application Id: 586655 **Chief Investigator A:** Dr Barbora de Courten **Application Year :** 2009

Additional Questions for the Applicant/General Comments

ONHMRC - GrantNet

ASSESSMENT REPORT FOR APPLICANT

Application Id: 586655 **Chief Investigator A:** Dr Barbora de Courten **Application Year :** 2009

Assessor 2

Scientific Quality

AGEs have been linked to beta cell deterioration and to insulin resistance via their effects on oxidative stress and also by activating inflammatory factors - the evidence linking these factors to deleterious effects is quite compelling. Whereas AGE formation has been mainly linked to endogenous states such as chronic hyperglycemia and oxidative stress, this project hypothesizes that dietary intake can also be a significant source of AGEs and literature support and some preliminary data is provided in support of this conjecture. It is claimed that even in subjects with presumably endogenous AGE formation, nonetheless additional dietary intake of AGEs can be deleterious and a clinical study is proposed to test this. The project is quite impressive and the trial has been carefully put together I believe the proposal is of a high standard. Some questions are listed separately.

Significance and Innovation

The project is innovative and its significance really boils down to how significant are dietary sources of AGEs in T2D and the metabolic syndrome. Based on the references supplied and the preliminary data this project seems quite well justified. It will apparently dovetail with other work of CIB investigating a rodent based model to firm up mechanisms (the animal-based preliminary data is apparently from this other project). There seems to be a strong base for the human studies proposed here and if successful the combined potential impact of the human studies here and the animal studies in the separate project will be considerable.

Track Record in Relation to Opportunity

Overall I feel that the applicants are capable of conducting these studies well. The CIA does not have all that many papers since setting up her lab in Melbourne but encouragingly her output seems to be improving with increasing recent output. In addition it is clear that there have been interruptions such as maternity leave and clinical training. There was not much evidence however of a significant international profile as yet. I am surprised that publications BDC1 and BDC2 are listed as having no citations. The CIB has an impressive publication record and should be a strength for the project. The CIC and CID are somewhat weaker but should be able to provide support in their respective areas of nutrition and statistical aspects. Some of CIC s publications are a bit obscure. Overall I would have preferred to see a little more hard evidence in each of the track record pages which tended to have sweeping statements such as my publications have been highly cited or unsupported statements in the Contribution to Knowledge sections.

Budget

The Budget is reasonable. I did however pose a question regarding the impression from the application that the one available glucose analyzer is a "rate limiting step". If this is indeed the case then perhaps a request for another analyzer would be justified if this would mean the project could be performed over a shorter period with less overall cost.

Application Id: 586655 **Chief Investigator A:** Dr Barbora de Courten **Application Year :** 2009

Questions for the Applicant

1 The likely success of the project depends quite critically on whether diet AGEs are significant contributors to the body AGE pool, particularly in subjects with elevated body AGEs. References 8 and 22 provide some good support but what else directly supports this in a compelling fashion?
2 While there may be a lot of evidence implicating AGEs in inflammatory processes, there is some uncertainty whether inflammatory processes are a cause or consequence of insulin resistance. Any comment?
3 It is stated that there is no Australian data on AGE in foods how certain are the applicants that a sufficient differential intake of AGEs can be set up to adequately test the hypothesis. Will this project be useful to establish Australian standards for AGE intake? How has the database in the US proved useful in a practical sense?
4 It is stated that with only one glucose analyser available for clamp studies, the maximum participant flow can be 16/year. While this perhaps conveniently spreads the project over the requested funding period, I really question whether this is the most efficient way to run it. Would it be more efficient to get another analyser and do the project over a shorter period?
5 A major component is the request for the AGE reader. It is stated that many AGEs have a characteristic fluorescence Are all relevant AGEs detectable?

Additional Questions for the Applicant/General Comments

Application Id: 586655 **Chief Investigator A:** Dr Barbora de Courten **Application Year :** 2009

Assessor 3

Scientific Quality

This is a well-designed research project, which will answer whether a reduction of dietary advanced glycation end products will improve insulin sensitivity and secretion in overweight individuals. The study protocol is extensive and the researchers have expertise in clinical trials and the molecular and biochemical analysis needed for the study to make it very productive. The main possible drawback is the difficulty in recruiting subjects given the extensive exclusion list but the fact that 8 subjects were recruited for the pilot study suggests that the researchers can achieve their recruitment timelines.

Significance and Innovation

This study is of major importance to human health. It takes the work of other groups looking at the role of dietary AGEs on insulin resistance and low level inflammation, which are significant to the development of diabetes, and then goes further in several innovative ways.
(1) It is a preventative medicine strategy as the test group are young healthy mildly-obese (and pre-diabetic) people.
(2) The high and low AGE diets reflect typical Australian meal selections which are easy for people to obtain and prepare and which avoids the problems of earlier studies where AGEs were either generated by overcooking the food or by addition of a specific AGE supplement.
(3) Evaluates the AGE content of typical Australian food.

If the hypothesis holds up as suggested by the preliminary data, this study could bring about a health revolution which could slow the march of type 2 diabetes by making a few simply changes to food preparation techniques.

Track Record in Relation to Opportunity

Dr Barbora de Courten' s track record is good, having published 11 papers in the last 5 years, in the mid-high impact range (1 cited >12 times).
A/Prof Josephine Forbes' track record is excellent, having 26 papers and 20 review articles (8 as first author) in mostly high impact journals (13 cited > 12 times).
Dr Karen Walker and A/Prof Maximilian de Courten have suitable experience for their respective parts in this project.
Together, the above researchers, have the knowledge and experience to carry out the proposed work.

Budget

Part-time salaries (50% for a research nurse, PSP3, and 30% for a research technician) are requested and suitable for the quantity of work involved. The direct research costs appear justified. The request for $39,500 for an AGE reader should be granted as it can measure non-invasively several fluorescent AGEs in skin and thus this study will not suffer from the problem of other dietary AGE studies which rely totally on one measure of AGE levels and uptake (i.e. CML elisa of serum and urine) and therefore may not reflect the true physiological status of the patient/volunteer.

Questions for the Applicant

Aim 1.
1. The preliminary data involved a high AGE vs low AGE diet of 61,388 and 9,532 kU AGE /day respectively, whilst the proposed research plan describes a high AGE diet of 108,000 kU AGE/ day. How will the differences in the high AGE diet with regard to AGE content be taken into account in the analysis of results?

2. The research plan uses a BMI cut off of greater and equal to 25 whilst the pilot study had more obese subjects with BMI of 34.1. Will this difference in obesity compromise the sample size estimation as differences with less obese subjects may be smaller?

Aim 2.
1. Based on previous work and the preliminary data, this study should show an association between AGE intake and (1) insulin sensitivity and (2) activation of inflammatory pathways but does not specifically address the aim that chronic activation plays an intermediary role in the association between AGE intake and insulin sensitivity.
2. What evidence is there that a mild reduction in inflammation markers (20%) would improve health outcomes in an obese or diabetic person?

Aim 3.
1. The background to the grant highlights the differences between Australian and American food items in respect to AGE levels, and yet the research plan and preliminary data use the American data for the diets in this study. Given the recruitment of suitable subjects for the study will be difficult, it would be better to do the AGE content analysis of the research plan meals at the beginning in case any modifications are required due to the Australian/ American differences, rather than concurrently as outlined in the timeline.

2. Whilst CIB has extensive experience of measuring CML in serum and urine samples, measuring CML in homogenates may produce difficulties not seen with serum and urine such as food insolubility, insensitivity to heat-treated foods and cross-reactivity with unrelated antigens. If problems are encountered here, how will they be addressed?

Additional Questions for the Applicant/General Comments

Assessor 1

Choice of inflammatory markers (IL-1β, IL-6, IL-10, hsCRP, TNFα, MIF, MCP-1, and adiponectin, NFKB activity)

The choice of the above listed inflammation markers for this study above other possible markers is based on many prospective studies (many published by CIA and colleagues) showing that these markers are associated prospectively with the development of diabetes.

The measurement of inflammatory markers as secondary endpoints

As stated by assessor 1, the inflammatory markers are secondary endpoints for this study. However, we anticipate that the design of this study, with patients as their own controls, may allow for the analysis of a change in the various inflammatory markers between the high and low AGE dietary periods, confirming their modulation by changes in AGE content within the circulation. A stated within the grant proposal, modulation of inflammatory markers by changing AGE dietary intake, has already been demonstrated in diabetic subjects.

Assessor 2

1. The contribution of dietary AGEs to the body's AGE pool

We now have evidence from another study, that differences in the cooking method alone are sufficient to affect serum levels of AGE CML. In that randomised cross-over study (Shahril et al, submitted for publication) each Mediterranean style diet consisted of the same Australian foods but cooked differently. Each diet was isocaloric with an identical macronutrient profile (43% energy as fat, 39% as carbohydrate) and consumed for four weeks. Under the slow cooked "Cretan style" meals (Diet A), serum CML fell (-50.3 ± 49.4 ng/mL), whilst rapidly cooked "high heat" style meals, serum CML rose (+37.8 ± 49.9, $P<0.05$). We therefore anticipate that we should see change in serum CML concentrations as consequence of our dietary interventions. In our study, we will also collect a 3-day record of the habitual diet before participants commence the study (i.e. before the "wash out" period). This record will link the habitual diet to baseline serum CML levels and together with the results of the study itself, should help indicate if some Australian dietary patterns are already excessively high in AGE content. Also because of the cross-over design, each patient acts as their own control.

2. Inflammation as a cause of insulin resistance

We believe that inflammation is one of the causes of insulin resistance rather than the reverse. CIA showed this for the first time in a prospective study in Pima Indians (Vozarova, Diabetes 2002) and this data was confirmed by 2 other independent studies in different populations. Moreover, CIA designed a study in Pima Indians which was recently published where an anti-inflammatory agent salsalate improved glucose tolerance and insulin resistance further supporting a causative role of insulin resistance in type 2 diabetes (ref BDC 9). Our preliminary animal studies indicate AGEs may interact with the inflammatory innate immune receptor RAGE, which is thought to be the most likely link between dietary AGE intake and insulin resistance.

3. US Data on AGE content of foodstuffs

The Assessor correctly indicates that there are yet no data on the AGE content of Australian foods and therefore we have used a US database. The US database indicates that high AGE foods are either foods that have been well browned during cooking (by grilling, roasting or frying) or are foods that are highly processed. Our proposed high AGE diets contains many foods from both categories and are designed to have at least a five-times higher AGE content than our low AGE diet.

We believe that generation of an Australian database of dietary AGE content is worthwhile in a practical sense (see also Assessor 3), since the US database has a particular utility for dietitians who are optimising diets for patients with inflammatory co-morbidities such as in diabetes and renal failure. Although we agree that it would be valuable to generate this Australian database before the completion of these studies, this is outside this application. However, we will endeavour to measure the AGE (CML) content of some more commonly consumed Australian foodstuffs at study commencement.

4. Availability of the Glucose Analyser

A second Analyser would cost approximately $ 80,000.- and could certainly speed up the study flow. To keep the budget low we did not include this capital expense but would appreciate the opportunity to obtain a second device. Other time critical processes of our study are the availability of the Cardiac Catheter Laboratories (in which the clamps are performed) and the availability of a trained endocrinologist (CIA) to perform the hyperinsulinaemic-euglycaemic clamp.

5. AGE reader and AGE detection

As suggested by the assessor, not all AGEs have fluorescent properties including carboxymethyllysine (CML). Skin associated fluorescence, however, has provided useful information about tissue concentrations of AGE fluorophores which have been shown to change within the post-prandial period (Ref 41). Therefore, the non-invasive nature and relative ease of this measurement are advantageous and may supersede the necessity for the assessment of circulating AGEs in blood samples in the future – a potential secondary outcome of our research.

Assessor 3

AGE content of high AGE diet

The study's dietitians are aiming to balance the weekly dietary AGE intake on the high AGE diet to approximately 61000kU of AGE/day. The daily AGE intake may vary, with some days (such as that represented in the example in the grant @ 108,000kU AGE) having an AGE intake more than 10 times than what we achieved under the low AGE diet. From this experience we are confident to achieve more than a 5-fold AGE difference between the diets, which exceeds what is currently published in the literature (3 times) and formed our original calculation.

BMI as a confounding factor

Assessor 3 states that the research plan uses a cut off ≥ 25 whilst the pilot study had more obese subjects (BMI-34). Will this difference in obesity compromise the sample size estimation as the differences in less obese subjects may be smaller?

To date, the mean and standard deviation obtained for BMI in all recruited subjects to date (n=12) is 29.5±6.4kg/m^2. Hence, the majority of subjects are within the range of overweight and class I obesity.

Effects of a 20% reduction in inflammatory parameters on health outcomes

It is impossible to predict whether a 20% reduction in inflammatory parameters will have meaningful effect on physiologically relevant outcomes such as insulin resistance in this study. Indeed measurement of inflammatory mediators is a secondary endpoint and the study is not designed/powered to assess the contribution of inflammatory markers *per se* to insulin resistance in healthy overweight individuals. However, the association of these inflammatory markers with modulation of dietary AGEs, which the study is powered to measure is highly relevant to this project and represents a mechanistic pathway which may explain the effects of dietary AGEs through the receptor RAGE, on insulin resistance.

Measurement of CML in homogenates

We agree that measurement of the AGE content of Australian homogenised foodstuffs, may be confounded by insoluble components or other as yet unidentified issues. CIB has two collaborators who are biochemists A/Prof Gerald Muench (University of Western Sydney) and Prof Michael Davies (Heart Research Institute) who will provide technical assistance if required.

Track record

We concur with assessors 1 and 3 that the track record of the team is very good, providing scientific, clinical, laboratory, dietary, statistical and public health experience to perform this kind of research. Although, track record of CIC is modest, the presence and supervision of the project by a dietician is essential.
